# Barriers to cervical cancer screening in Guatemala: a quantitative analysis using data from the Guatemala Demographic and Health Surveys

**DOI:** 10.1007/s00038-019-01319-9

**Published:** 2019-12-14

**Authors:** Anna Gottschlich, Pamela Ochoa, Alvaro Rivera-Andrade, Christian S. Alvarez, Carlos Mendoza Montano, Claudia Camel, Rafael Meza

**Affiliations:** 1 Department of Epidemiology, School of Public Health, University of Michigan, Ann Arbor, MI USA; 2 PRA Health Sciences, Guatemala City, Guatemala; 3 Research Center for the Prevention of Chronic Diseases, Institute of Nutrition of Central America and Panama-INCAP, Guatemala City, Guatemala; 4 Division of Cancer Epidemiology and Genetics, National Cancer Institute, Bethesda, MD USA; 5 Guatemala Ministry of Health and Social Assistance (MSPAS), Guatemala City, Guatemala

**Keywords:** Cervical cancer, Screening, Healthcare barriers, Indigenous communities

## Abstract

**Objectives:**

Examine the association between commonly reported barriers to health care, including discordant spoken languages between patients and providers, and reported previous cervical cancer screening.

**Methods:**

Data from the nationally representative Guatemala National Maternal and Child Health Survey from the Demographic and Health Surveys Program were used to explore associations between barriers and screening rates nationwide and in high-risk populations, such as rural and indigenous communities. Negative binomial regressions were run accounting for survey sample weights to calculate prevalence ratios.

**Results:**

64.0%, 57.5% and 47.5% of women reported ever screening, in the overall, indigenous, and rural populations, respectively. Overall, never screened for cervical cancer was associated with the following health barriers: needing permission, cost, distance, not wanting to go alone, and primary language not spoken by health providers, even after adjustment for age, ethnicity, and literacy.

**Conclusions:**

Offering screening programs alone is not enough to reduce the burden of cervical cancer in Guatemala. Measures need to be taken to reduce barriers to health care, particularly in rural areas, where screening rates are lowest.

**Electronic supplementary material:**

The online version of this article (10.1007/s00038-019-01319-9) contains supplementary material, which is available to authorized users.

## Introduction

Cervical cancer is the fourth most commonly diagnosed cancer in women worldwide, with 85% of cases in low- and middle-income countries (LMICs) (Jemal et al. 2010). The burden of this disease differs vastly between LMICs and high-income countries (HICs), where rates of cervical cancer are low. In Central America, cervical cancer is the second most common cause of cancer death in women (Soneji and Fukui 2013; Ferlay et al. 2019), and in Guatemala rates of cervical cancer mortality are six times that in the USA (ASMRs: 11.7 death vs. 1.9 deaths per 100,000 person-years, respectively) (International Agency for Research on Cancer 2018). This discrepancy begs the question of why women are dying from a disease that is mostly avoidable through screening practices commonly and effectively implemented in HICs.

Latin American countries experience many challenges that directly affect mortality rates from cervical cancer. Screening rates remain low, even in countries with national screening programs, perhaps due to cultural, logistical, and cost barriers (Soneji and Fukui 2013). Additionally, limited supply of highly trained healthcare providers and laboratory technicians, insufficient evaluation and follow-up of results, and lacking availability of access to screening contribute to low rates, limiting progress in the reduction of cervical cancer (Soneji and Fukui 2013). Fragmented health systems cause delays in diagnostic workup, often resulting in advanced tumors that necessitate expensive diagnostic procedures and treatments (Strasser-Weippl et al. 2015), which are not readily available. Finally, access to high-cost drugs and implementation of novel early detection and treatment technologies is limited.

The Ministry of Health (MOH) of Guatemala has worked to implement effective screening and treatment programs since the 1950s, and the National Reproductive Health Program (PNSR) has been strengthening the training and infrastructure for these programs since its creation in 2000 (Ministerio de Salud Pública y Asistencia Social 2014). According to the MOH, the public health system in Guatemala has 27 cytology laboratories, 7 pathologists, 21 cytotechnologists, 32 colposcopes, 33 cryotherapy units, 9 thermocoagulation units, and 11 electrosurgery units (de León et al. 2014; Ministerio de Salud Pública y Asistencia Social 2014; Camel 2018). However, even with infrastructure in place, studies show that less than half of women in Guatemala report ever been screened for cervical cancer (Chary and Rohloff 2014). In Guatemala, free health care is a constitutional right, and the MOH offers screening at no cost (Chary and Rohloff 2014). However, annual screening coverage by the MOH is only 12–18%, while total screening coverage is around 40% (Chary and Rohloff 2014). The World Health Organization recommends 80% coverage for successful national screening programs (Ferlay et al. 2012). Women also have challenges receiving screening results and accessing follow-up care (Holme et al. 2017; Austad et al. 2018; Im et al. 2018), and, in some cases, the quality and sensitivity of Pap smears have been called into question (Im et al. 2018).

Lack of access to screening in LMICs is a main reason for higher rates of cervical cancer (Austad et al. 2018; Im et al. 2018). Several studies have assessed barriers to cervical cancer screening in the USA, England, Serbia, Mexico, and other parts of Latin America (Lee 2000; Watkins et al. 2002; Agurto et al. 2004; Markovic et al. 2005; Waller et al. 2009). Barriers to screening include practical factors such lack of knowledge (Agurto et al. 2004; Markovic et al. 2005; Andreassen et al. 2018), prohibitive costs (Lee 2000), and difficulty accessing the healthcare system (Lee 2000; Watkins et al. 2002; Agurto et al. 2004; Markovic et al. 2005; Waller et al. 2009), as well as emotional factors such as embarrassment (Lee 2000; Watkins et al. 2002; Agurto et al. 2004), fear of pain (Lee 2000; Agurto et al. 2004), and socio-cultural health beliefs (Lee 2000; Watkins et al. 2002). In response to these, increases in screening education, taking into account cultural beliefs and local context, and increased accessibility to health services have been proposed.

However, there have been few screening barrier studies in primarily indigenous populations (Chary and Rohloff 2014; Austad et al. 2018), who tend to be underserved in many aspects, including health care (Chary et al. 2017), and who constitute approximately half the population of Guatemala. Furthermore, there are 24 spoken languages in Guatemala (Ministerio de Salud Pública y Asistencia Social 2014), and it has been reported that lack of concordant languages spoken by communities and their doctors creates additional barriers to healthcare services (Avila et al. 2015). Yet, there is a gap in the literature exploring the association between discordant languages and participation in cervical cancer screening. To date, it remains unclear whether barriers to cervical screening for non-indigenous populations described above are similar among indigenous populations, who are disproportionately burdened by this disease. This paper used data from the 2014 to 2015 Guatemala National Maternal and Child Health Survey (ENSMI) (USAID 2018) to investigate factors associated with screening for cervical cancer in subpopulations throughout Guatemala. Specifically, we assessed the effect that access barriers to health care, including not speaking the same language as community health providers, have on the rates of ever and recent cervical cancer screening among the general, rural, and indigenous populations.

## Methods

### Data

ENSMI is a nationally representative cross sectionally designed survey, carried out within the framework of the USAID Demographic and Health Surveys (DHS) program. The main purpose of the DHS program is to assist countries in developing, implementing, and analyzing nationally representative household surveys that provide data on population and health indicators. DHS provided technical assistance, supplies, and equipment to support ENSMI, which was implemented by the Institute of Nutrition for Central American and Panama (INCAP) (MSPAS et al. 2017). Further references to the survey will use “DHS/ENSMI” to avoid confusion.

The most recent fieldwork went from October 2014 to June 2015. The National Institute of Statistics (INE) used a multi-stage stratified selection procedure in which approximately 36 sectors were selected randomly (proportionate to the overall number of urban and rural sectors defined by INE) from each of Guatemala’s 22 departments. Then, approximately 26 homes were selected in each sector, creating a sample from the frame of the entire country that can be reasonably assumed as representative at the national, residential (urban vs rural) and regional (department) levels. Of the 22,308 households selected for participation, 21,383 (95.9%) were successfully recruited, and from these, 25,914 women and 11,145 men responded to the survey. Female and male participants were 15–49 and 15–59 years old, respectively (USAID 2018).

### Variable creation

The complete dataset was analyzed, representative of the entire country, and then restricted to rural and indigenous populations. DHS/ENSMI recorded participants as residing in rural or urban areas according to criteria defined by INE (Instituto Nacional de Estadıstica 2011). In this classification, each department has both rural and urban areas. To create the rural subpopulation, we included persons living in rural areas of any department, while persons living in urban areas were excluded. The DHS/ENSMI asked participants to define their ethnicity, given the following choices: Ladino (persons of mixed ancestry), Maya (persons of indigenous descent), Xinca (persons of indigenous descent), Garifuna (persons of Afro-Caribbean descent), or other. Other counts were less than 1% of the data and thus were dropped from analyses. To create the indigenous subpopulation, we included persons who responded that they were either Maya, Garifuna, or Xinca, and excluded Ladinos. Survey sampling weights, available from DHS, were used in all analyses. Finally, analyses were subset to women in the age range of 25–49 to attempt to mimic both the Guatemalan National Guide for Testing and Treating Precancerous Lesions to Prevent Cervical Cancer (de León et al. 2014) and the National Cancer Prevention Plan (Ministerio de Salud Pública y Asistencia Social 2014) guidelines, which both recommend cervical cancer screening for women ages 25–54. Unfortunately, DHS/ENSMI does not survey women over the age of 49, and so we were unable to assess women in the 50–54 age range. While only women who have been sexually active are at risk for cervical cancer, we decided to include all women in this age range, because neither guideline excludes those with no sexual history, and since sexual history is self-reported.

The main outcome explored was prior cervical cancer screening. We defined this as screening that was understood and remembered by the woman receiving it. Thus, women who responded “No” to having heard of cervical cancer or screening were included in the “Never Screened” group, even though they were **not** directly asked the follow-up question about ever been screened. Women who responded “Yes” to having heard of cervical cancer or screening and then “No” to ever screening were also included in the “Never Screened” group. Finally, women who responded “Yes” to both having heard of cervical cancer or screening and to prior screening were included in the “Ever Screened” group. USAID’s Health Finance and Governance project used a similar definition in their 2015 Health Systems Assessment Report, including those who had never heard of screening in the “Never Screened” group (Avila et al. 2015). This binary ever-screened variable was used as an outcome to investigate predictors and barriers to screening across subpopulations.

According to both previously mentioned screening guidelines, (de León et al. 2014; Ministerio de Salud Pública y Asistencia Social 2014), women with a prior negative screen should continue cytological screening approximately every three years. Thus, we also explored prevalence of screening within this timeframe, to measure compliance with frequency recommendations. The survey asked women their current age as well as age at last Pap. Using this data, we created an indicator to identify cervical cancer screening in the past three years. The percent of “up-to-date” women across population groups were then calculated and compared to the percent of women reporting ever-screening.

Predictor variables explored included potential barriers to screening. The DHS/ENSMI asked women about perceived barriers to health care. These barriers included needing permission from family to access care, the cost of the care, the distance to the facility, and not having anyone to travel with to a facility. Each barrier was constructed as a binary variable (yes/no). Additionally, we created a language barrier variable as a potential predictor. The DHS/ENSMI asked women the primary language spoken in their home and what languages were spoken by health professionals in local facilities. A binary variable was created to identify if there was a match between the participant’s primary language and any language spoken at local facilities.

Finally, age, ethnicity, and education (none, primary, secondary, or higher) were used to adjust regression models. Other demographic variables were explored to compare women with prior screening to those with no screening, including a wealth index developed by the DHS. This index is produced from a score created using interviewer-observed household characteristics (such as material of the house, source of water, number of people per room, and electronic items in the house) and is thus less susceptible to responder misclassification than self-reported variables such as income and education (MSPAS et al. 2017).

Missingness was less than 0.05% for all variables used in analyses.

### Statistical analyses

Women with prior screening were compared to those with none, and binary and categorical variables were compared using proportion and Chi-square tests, respectively. Continuous variables were tested using a *t* test to compare means. Alpha was set at 0.05, and unweighted count, percentage of subgroup, standard deviation, and *p* value are shown in relevant tables.

Additionally, prevalence ratios of never screened were modeled using multivariate negative binomial regression with robust variance using reported barriers to health care, including language, as predictors. Crude models were adjusted for age, ethnicity, and education: factors previously shown in the literature to be associated with screening (Soneji and Fukui 2013).

Sensitivity analyses were conducted to assess the impact of our definition of prior screening. First, models were rerun considering only those participants who had heard of screening and thus were explicitly asked whether they had previously screened. Then, models were rerun, subset to only those who reported prior sexual activity, and were thus truly at risk for cervical cancer according to their self-reported history.

Data cleaning and statistical analyses were conducted using the R statistical software version 3.4.4.

## Results

The 2014–2015 DHS/ENSMI recruited 25,914 women of ages 15–49. Our analyses used data from the subset of 15,317 women of ages 25–49 (Guatemala *N* = 15,317, Rural *N* = 8399, Indigenous *N* = 5728). The weighted average age was approximately 36 years old across all populations. In the overall population, 63.7% of women reported prior screening. This number decreased in the rural and indigenous populations, with only 57.5% and 47.5% reporting prior screening, respectively (Table 1).

**Table 1:** Distribution of demographic variables: National Survey of Maternal and Child Health, Guatemala, 2014–2015

	Guatemala					Rural					Indigenous				
	Ever screened^B^ (*N* = 9660, 63.7%)		Never screened^B^ (*N.* = 5640, 36.3%)		*P*	Ever screened^B^ (*N* = 4852, 57.5%)		Never screened^B^ (*N* = 3539, 42.5%)		*P*	Ever screened^B^ (*N* = 2752, 47.5%)		Never screened^B^ (*N* = 2970, 52.5%)		*P*
	%/mean (SD)^A^	*N*	%/mean (SD)^A^	*N*		%/mean (SD)^A^	*N*	%/mean (SD)^A^	*N*		%/mean (SD)^A^	*N*	%/mean (SD)^A^	*N*	
Age	36.38 (0.087)	NA	33.49 (0.109)	NA	< 0.0010	36.11 (0.125)	NA	33.83 (0.139)	NA	< 0.001	36.49 (0.149)	NA	34.26 (0.143)	NA	< 0.00100
Urban	52.16 (0.01)	4808	38.05 (0.012)	2101	< 0.0010	–	–	–	–	–	35.41 (0.019)	1073	30.31 (0.02)	962	0.01763
Ethnicity					< 0.0010					< 0.001					0.49340
Maya	28.4 (0.012)	2663	55.78 (0.015)	2921		38.14 (0.019)	1609	62.79 (0.019)	1973		97.66 (0.004)	2663	99.02 (0.002)	2921	
Ladino	70.87 (0.012)	6900	43.54 (0.015)	2661		60.66 (0.019)	3167	36.45 (0.019)	1523		–	–	–	–	
Garifuna	0.03 (0.0001)	4	0.04 (0.0002)	4		0.019 (0.0002)	1	0.03 (0.0002)	2		0.12 (0.001)	4	0.01 (0.0004)	4	
Xinca	0.06 (0.001)	85	0.05 (0.001)	45		1.1 (0.002)	69	0.56 (0.002)	33		2.22 (0.004)	85	0.09 (0.002)	45	
Education					< 0.0010					< 0.001					< 0.00100
None	15.86 (0.006)	1471	30.16 (0.011)	1646		23.54 (0.01)	1047	36.79 (0.014)	1254		31.36 (0.014)	788	41.84 (0.016)	1196	
Primary	49.3 (0.008)	4867	46.69 (0.009)	2687		60.15 (0.011)	2986	51.66 (0.013)	1856		52.67 (0.014)	1494	45.68 (0.014)	1380	
Secondary	26.41 (0.007)	2483	17.13 (0.007)	972		14.3 (0.009)	700	9.06 (0.006)	350		13.02 (0.01)	375	9.99 (0.008)	322	
Higher	8.43 (0.005)	839	6.01 (0.004)	335		2.01 (0.003)	119	1.43 (0.005)	79		2.96 (0.004)	95	2.49 (0.004)	72	
Literate	83.45 (0.006)	8103	67.1 (0.012)	3851	< 0.0010	74.6 (0.101)	3699	59.04 (0.014)	2156	< 0.001	67.62 (0.013)	1929	54.35 (0.017)	1665	< 0.00100
Wealth index					< 0.0010					< 0.001					0.01360
Poorest	11.42 (0.005)	1136	25.98 (0.012)	1424		21.2 (0.011)	1015	36 (0.015)	1232		24.49 (0.014)	627	35.72 (0.018)	998	
Poorer	14.37 (0.005)	1476	23.19 (0.009)	1324		24.27 (0.009)	1200	29.29 (0.013)	1038		23.51 (0.011)	647	27.36 (0.013)	815	
Middle	18.82 (0.007)	1894	20.79 (0.008)	1226		26.86 (0.01)	1280	21.67 (0.011)	795		21.8 (0.013)	617	20.74 (0.012)	656	
Richer	25.28 (0.008)	2398	17.61 (0.008)	986		19.61 (0.009)	939	9.96 (0.008)	361		18.07 (0.011)	540	12.16 (0.009)	364	
Richest	30.11 (0.009)	2756	12.42 (0.007)	680		8.06 (0.007)	418	3.07 (0.005)	113		11.5 (0.009)	321	4.01 (0.005)	137	
Currently married/united	81.19 (0.005)	7855	62.75 (0.009)	3504	< 0.0010	85.66 (0.006)	4139	68.28 (0.011)	2378	< 0.001	86.47 (0.009)	2373	68.73 (0.012)	2019	< 0.00100
Ever married/united	95.82 (0.003)	9272	73.98 (0.008)	4138	< 0.0010	97.37 (0.003)	4728	79.24 (0.009)	2763	< 0.001	97.22 (0.004)	2680	79.04 (0.01)	2310	< 0.00100
Age at marriage	19.11 (0.062)	NA	19.04 (0.089)	NA	0.5052	18.39 (0.073)	NA	18.79 (0.099)	NA	< 0.001	18.57 (0.105)	NA	18.74 (0.113)	NA	0.19150

Population has been subset to only include women aged 25 or older to match Guatemalan screening guidelines

^A^Percentages, means, and standard deviations are weighted based on DHS-provided weights

^B^Ns are unweighted to show the relative contribution

### Demographics

Demographics, stratified by screening status, are shown in Table 1. Women who reported prior screening were older across populations (Guatemala: 36.38 vs 33.49, *p* < 0.001; Rural: 36.11 vs 33.38, *p* < 0.001; Indigenous: 36.49 vs 34.26, *p* < 0.001). Women in the overall and indigenous groups living in urban areas were more likely to have screened than those in rural areas, although this difference was smaller in the indigenous population (Guatemala: 52.16% vs 38.05%, *p* < 0.001; Indigenous: 35.41% vs 30.31%, *p* < 0.001). Non-indigenous women in the overall and rural populations were more likely to have screened than indigenous, although again, the difference was smaller in the rural than overall population. Women with higher education, who were literate, and who were in higher wealth index quintiles were more likely to have screened across populations. In the rural and indigenous populations, there were overall fewer women with higher education and wealth, and fewer literate women. Finally, women who were currently or ever married were more likely than those who were not to report screening.

### Sexual and health history

Women who reported prior screening also reported a younger age at sexual debut (Guatemala: 18.23 vs 18.79; Rural: 17.66 vs 18.39; Indigenous: 17.94 vs 18.36), a higher number of lifetime sexual partners (Guatemala: 1.56 vs 1.44; Rural: 1.36 vs 1.11; Indigenous: 1.34 vs 1.06), and more children than those who reported never screening (Table 2). When comparing all of Guatemala to rural and indigenous groups, more sexual partners were reported overall, but more children and younger ages for birth of first child were reported by rural and indigenous women. Women who reported visiting health facilities in the last year were more likely than those who did not to report screening (Guatemala: 72.89% vs 60.79%; Rural: 75.21% vs 62.84%; Indigenous: 73.00% vs 59.15%). Across all populations, age at sexual debut was approximately 18 years old, similar to the rates in the USA (Centers for Disease Control and Prevention 2017), which has similar screening guidelines as Guatemala.

**Table 2:** Distribution of sexual- and health-related variables: National Survey of Maternal and Child Health, Guatemala, 2014–2015

	Guatemala					Rural					Indigenous				
	Ever Screened^B^ (*N* = 9660, 63.7%)		Never Screened^B^ (*N* = 5640, 36.3%)		*P*	Ever Screened^B^ (*N* = 4852, 57.5%)		Never Screened^B^ (*N* = 3539, 42.5%)		*P*	Ever Screened^B^ (*N* = 2752, 47.5%)		Never Screened^B^ (*N* = 2970, 52.5%)		*P*
	%/mean (SD)^A^	*N*	%/mean (SD)^A^	*N*		%/mean (SD)^A^	*N*	%/mean (SD)^A^	*N*		%/mean (SD)^A^	*N*	%/mean (SD)^A^	*N*	
Age	36.38 (0.087)	NA	33.49 (0.109)	NA	< 0.0010	36.11 (0.125)	NA	33.83 (0.139)	NA	< 0.0010	36.49 (0.149)	NA	34.26 (0.143)	NA	< 0.00100
Sexually active	99.9 (0.0004)	9645	82.46 (0.007)	4599	< 0.0010	99.93 (0.0003)	4847	85.36 (0.008)	2978	< 0.0010	99.82 (0.0009)	2746	84.65 (0.01)	2478	< 0.00100
Age at first sex	18.23 (0.068)	NA	18.79 (0.106)	NA	< 0.0010	17.66 (0.096)	NA	18.39 (0.13)	NA	< 0.0010	17.94 (0.13)	NA	18.36 (0.13)	NA	0.00990
Partners last year	0.87 (0.017)	NA	0.64 (0.009)	NA	0.0658	0.85 (0.007)	NA	0.66 (0.011)	NA	< 0.0010	0.84 (0.009)	NA	0.65 (0.012)	NA	< 0.00100
Lifetime partners	1.56 (0.04)	NA	1.14 (0.035)	NA	0.0004	1.36 (0.02)	NA	1.11 (0.044)	NA	0.1923	1.34 (0.075)	NA	1.06 (0.027)	NA	0.06571
No. of children	3.46 (0.034)	NA	2.97 (0.058)	NA	< 0.0010	4.08 (0.056)	NA	3.36 (0.071)	NA	< 0.0010	4.28 (0.067)	NA	3.37 (0.078)	NA	< 0.00100
Age at first pregnancy	20.16 (0.058)	NA	20.31 (0.081)	NA	0.1009	19.5 (0.066)	NA	20.09 (0.088)	NA	< 0.0010	19.75 (0.092)	NA	20.15 (0.104)	NA	0.00120
Visited health facility in last 12 mo.	72.89 (0.006)	7093	60.79 (0.009)	3438	< 0.0010	75.21 (0.009)	3654	62.84 (0.012)	2246	< 0.0010	73 (0.009)	1994	59.15 (0.013)	1725	< 0.00100

Population has been subset to only include women aged 25 or older to match Guatemalan screening guidelines

^A^Percentages, means, and standard deviations are weighted based on DHS-provided weights

^B^Ns are unweighted to show the relative contribution

### Knowledge of cervical cancer

Table 3 shows the distribution of knowledge of cervical cancer. Knowledge of cervical cancer is high across all populations but is lower among rural and indigenous women (overall 91.6%, rural 87.8%, indigenous 82.6%). Among those who had heard of cervical cancer, those who had heard of screening were similarly high, but again lower among indigenous women.

**Table 3:** Distribution of cervical cancer knowledge and prior screening variables: National Survey of Maternal and Child Health, Guatemala, 2014–2015

	Guatemala (*N* = 15,317)		Rural (*N* = 8399)		Indigenous (*N* = 5728)	
	% (SD)^A^	*N*^B^	% (SD)^A^	*N*^B^	% (SD)^A^	*N*^B^
Heard of cervical cancer	91.6 (0.0047)	14,036	87.8 (0.0072)	7422	82.6 (0.0105)	4750
If heard of CC, heard of CC exam	95.33 (0.0025)	13,409	94.59 (0.0038)	7060	92.5 (0.0052)	4414
If heard of CC, but not heard of CC exam, heard of Pap	88.33 (0.0146)	545	84.5 (0.0221)	296	80.85 (0.0244)	263
Ever screened	63.7 (0.007)	9660	57.5 (0.01)	4852	47.5 (0.0114)	2752
Screened past 3 years (among total population)	50.0 (0.007)	7552	45.2 (0.01)	3803	36.1 (0.0108)	2098

Population has been subset to only include women aged 25 or older to match Guatemalan screening guidelines

^A^Percentages and standard deviations are weighted based on DHS-provided weights

^B^Ns are unweighted to show the relative contribution

Interestingly, women in the overall population who had heard of screening were more likely to have also received screening than women in the rural and indigenous populations (70% overall, 66% rural, 58% indigenous) (Appendix Table A1).

### Screening

As mentioned above, rates of prior screening were 47.5%, 57.5% and 63.7% for the indigenous, rural, and overall populations, respectively. However, in the overall population, only 50.1% of women reported screening within the last three years (Table 3). In the rural and indigenous populations, the percent of women reporting screening within the last three years was even lower (45.3% and 36.1% in the rural and indigenous populations, respectively).

### Barriers

Across all populations, cost was the most reported barrier (over 60% in each group reported cost as a barrier), followed by distance (over 35% in each group), not wanting to go alone, and finally needing permission (Table 4). Women who had never screened for cervical cancer were more likely to report each of the barriers across all populations (for example, in the overall population 60.8% reported cost as a barrier among screeners, while 67.5% of non-screeners reported this barrier). More women in the rural and indigenous populations reported barriers than in the overall population: needing permission was more common in the indigenous population than rural, while distance to the health facility was more common in the rural population than indigenous. Money and not wanting to go alone were similarly reported across the groups.

**Table 4:** Distribution of barriers to medical care variables: National Survey of Maternal and Child Health, Guatemala, 2014–2015

	Guatemala					Rural					Indigenous				
	Ever Screened (*N* = 9660, 63.7%)		Never Screened (*N.* = 5640, 36.3%)		*P*	Ever Screened (*N* = 4852, 57.5%)		Never Screened (*N* = 3539, 42.5%)		*P*	Ever Screened (*N* = 2752, 47.5%)		Never Screened (*N* = 2970, 52.5%)		*P*
	% (SD)^A^	*N*^B^	% (SD)^A^	*N*^B^		% (SD)^A^	*N*^B^	% (SD)^A^	*N*^B^		% (SD)^A^	*N*^B^	% (SD)^A^	*N*^B^	
Permission	13.4 (0.004)	1235	20.3 (0.007)	1055	< 0.001	14.2 (0.007)	631	21.8 (0.01)	715	< 0.00100	15.9 (0.009)	425	22.8 (0.01)	645	< 0.00100
Money	60.8 (0.008)	5864	67.5 (0.008)	3773	< 0.001	69 (0.009)	3312	71.6 (0.009)	2499	0.02900	70.2 (0.012)	1918	72.6 (0.011)	2167	0.08789
Distance	35.8 (0.008)	3442	45.9 (0.011)	2516	< 0.001	50 (0.013)	2419	56.1 (0.013)	1954	0.00013	46.8 (0.016)	1236	51.8 (0.016)	1504	0.01018
Travel alone	22.4 (0.006)	2133	33 (0.009)	1815	< 0.001	27.9 (0.009)	1331	36.8 (0.012)	1262	< 0.00100	28.1 (0.013)	747	36.1 (0.014)	1045	< 0.00100
Language	10.7 (0.006)	1001	28 (0.014)	1421	< 0.001	16.5 (0.011)	700	33.5 (0.018)	1016	< 0.00100	35.9 (0.017)	983	49.3 (0.017)	1411	< 0.00100

Population has been subset to only include women aged 25 or older to match Guatemalan screening guidelines

^A^Percentages and standard deviations are weighted based on DHS-provided weights

^B^Ns are unweighted to show the relative contribution

Women who had a doctor in their local health facility who spoke their primary language were statistically significantly more likely to have received screening than those who did not. Among indigenous women, of those who had ever screened, 64% had a doctor who spoke their primary language, while only 50% of women who had never screened had the same.

Results from multivariate analyses can be found in Table 5. The prevalence of prior screening was lower given any of the barriers, remaining significant, or nearly significant, even after adjustment for age, ethnicity, and education. Even though needing permission and not wanting to go alone were less commonly reported than money or distance, they were more strongly related to screening status. The prevalence of those who had never screened for women who reported needing permission in the overall population, the rural, and the indigenous subpopulation were 1.22 (95% CI 1.15, 1.29), 1.21(95% CI 1.13, 1.30), and 1.18 (95% CI 1.10, 1.27) times that of women who did not report needing permission, respectively. Similarly, for not wanting to go alone, the prevalence of no prior screening was 1.22 (95% CI 1.16, 1.28), 1.17 (95% CI 1.10, 1.24), and 1.15 (95% CI 1.08, 1.22) times that of women not reporting this barrier, respectively. The prevalence of no prior screening among women who did not have access to a doctor who spoke their primary language was between 1.19 and 1.25 times the prevalence than those who did, across populations.

**Table 5:** Prevalence ratios of never prior cervical cancer screening: National Survey of Maternal and Child Health, Guatemala, 2014–2015

	Guatemala		Rural		Indigenous	
	PR	95% CI	PR	95% CI	PR	95% CI
Permission	1.22	1.15, 1.29	1.21	1.13, 1.30	1.18	1.10, 1.27
Money	1.08	1.03, 1.14	1.04	0.98, 1.11	1.05	0.98, 1.12
Distance	1.10	1.05, 1.16	1.09	1.02, 1.16	1.07	0.99, 1.14
Travel alone	1.22	1.16, 1.28	1.17	1.10, 1.24	1.15	1.08, 1.22
Language	1.23	1.16, 1.31	1.19	1.10, 1.28	1.25	1.18, 1.32

Population has been subset to only include women aged 25 or older to match Guatemalan screening guidelines

Prevalence ratios modeled using multivariate negative binomial regression with robust variance

Models additionally adjusted for age, ethnicity (Maya, Ladino, Garifuna, Xinca), and education level (none, primary, secondary, higher)

Counts for each variable can be found in Table 4

The results of both sensitivity analyses are provided in the Appendix (Tables A2 and A3). Results from both analyses are consistent with our main analysis.

## Discussion

This study has three important findings. First, there is a high rate of knowledge of cervical cancer and screening among women in Guatemala; however, rates of reported ever screening are still quite low. Women who are from rural or indigenous populations are less likely to have screened, even if they have heard of screening, than their country-wide counterparts. Compliance rates are even lower, and unfortunately recent screening has not increased meaningfully since the last Guatemalan DHS, conducted in 1999 (32.8% report screening in the 2014–2015 survey versus 26.5% in the 1999 survey) (Soneji and Fukui 2013). Thus, providing knowledge alone is clearly not sufficient to improve cervical cancer prevention in this setting. Second, reported barriers to health care, including needing permission, not having the required cost for care, not being able to travel the distance to health facilities, or not wanting to go alone, are significantly associated with no prior screening. These barriers need to be addressed to improve screening rates in Guatemala. Finally, women who do not speak the same language as their local health providers are less likely to have had screening. In countries like Guatemala, where many languages are spoken, additional efforts should be made to reduce language barriers in healthcare settings.

Additionally, our analyses showed that a variety of demographic factors are predictive of prior screening. Living in an urban area versus rural, identifying as non-indigenous versus indigenous, being literate and more highly educated, and being in a higher wealth index were all positively predictive of prior screening.

This study used the nationally representative Guatemala 2014–2015 DHS/ENSMI survey, which is the first DHS survey in Guatemala since the late 1990s. This dataset allows for a better understanding of accessibility (or lack thereof) of health care, and in particular as shown here, cervical cancer screening, among different populations around the country. The DHS is a long-standing and rigorous survey, with a larger sample size and more in-depth questionnaires than other smaller studies could manage. Thus, the DHS provides a reliable dataset for assessing screening access, which includes standard questions that are used in the same survey in other countries around the world.

However, this study has some limitations. The sampling design was based on the most recent Guatemalan census, which was conducted in 2002, a decade before survey planning began, leading to sampling decisions being made using 10-year-old data, which could lead to some biases as the urban population in Guatemala has been growing (Central Intelligence Agency 2019). Additionally, much of the data used in these analyses are self-reported. As is recognized with reproductive care and healthcare access questions, there is a high possibility of misreporting, due to recall and desirability bias (Stuart and Grimes 2009; Gonsalves and Hindin 2017). For example, screening rates could either be over- or underestimated depending on whether there was a social desirability effect, which caused women to over-report screening, or a recall bias effect where women forgot or did not understand the meaning of getting screened, causing under-reporting. Finally, since participants self-reported the languages are spoken at clinics, we may be missing languages that participants were unaware were also spoken, although it is unlikely that they would not report their own. Independently, we believe that, despite this limitation, the correlation between perceived languages spoken and screening practices is an important finding in itself.

When the DHS released their final report of these data, they used all women from ages 15–49 to investigate access to cervical cancer screening. However, both Guatemala screening guidelines recommend that only women ages 25–54 should get screened for cervical cancer, and so we choose to only analyze women in this age group (truncated at age 49). Because of this, our reported screening and knowledge rates are slightly higher than those reported by the DHS because women ages 15–24 (who were excluded in our analyses) are generally not screened for and are less knowledgeable about cervical cancer (heard of Pap screening: 84.0% reported by DHS versus 88.3% reported in Table 3; prior screening, given knowledge of Pap: 49.8% reported by DHS and 69.9 reported in Table A1).

Many studies report ever-screened figures when discussing cervical cancer screening, but fewer look at up-to-date screening, given country-specific recommendations. Our analyses showed, similar to other studies done in Guatemala (Murchland et al. 2019; Gottschlich et al. 2017), that around 60% of women report being screened for cervical cancer at some point in their life, however, as few as 40–50% are up-to-date on their screening.

With respect to barriers to health care, our findings are similar to the literature (Lee 2000; Watkins et al. 2002; Agurto et al. 2004; Markovic et al. 2005; Waller et al. 2009), even among the less commonly investigated indigenous populations. Other studies from Guatemala cite distance, cost, and lack of social support as barriers to accessing health care (Holme et al. 2017; Austad et al. 2018; Im et al. 2018). Similarly, this study found that cost and distance were the most commonly reported barriers to screening in all populations, including indigenous, and these, as well as needing permission and not wanted to go alone, were all statistically significantly associated with prior screening. These conclusions, along additional finding that language discordance is associated with lack of screening, can support practitioners and policy makers as they develop plans to improve cervical screening, and general healthcare delivery, in Guatemala.

Unfortunately, better access to screening programs alone is not enough to reduce the burden of cervical cancer in LMICs, like Guatemala. Additional studies are needed to understand the barriers and facilitators to not only getting screened, but also receiving the results of a screening test, and accessing any needed follow-up care and eventual treatment. Nonetheless, our study provides an updated assessment of the patterns of access to screening and the most salient barriers among highly at-risk groups in Guatemala, which is a critical step to evaluate the chain of events required for successful cervical cancer prevention programs.

## Electronic supplementary material

Below is the link to the electronic supplementary material.

Supplementary material 1 (DOCX 20 kb)
