# Supplementary material for: Barriers to cervical cancer screening in Guatemala: a quantitative analysis using data from the Guatemala Demographic and Health Surveys
Source: Int J Public Health. 2019 Dec 14;65(2):217–26. doi: 10.1007/s00038-019-01319-9 (PMC7049547; doi:10.1007/s00038-019-01319-9)
Supplement: Supplementary file 1 — Supplementary material 1 (DOCX 20 kb) [file 38_2019_1319_MOESM1_ESM.docx]

**Barriers to Cervical Cancer Screening:**

**A Quantitative Analysis Using Data from the Guatemala Demographic and Health Surveys**

Anna Gottschlich^1^, Pamela Ochoa^2^, Alvaro Rivera-Andrade^3^, Christian S. Alvarez^1,4^, Carlos Mendoza Montano^3^, Claudia Camel^5^, Rafael Meza^1^

**Supplementary Electronic Appendix**

Table A1 – Prior Knowledge and Screening Reported Among Populations

Table A2 – Sensitivity analysis – Prevalence ratios excluding those who have never heard of cervical cancer testing

Table A3 – Sensitivity analysis – Prevalence ratios excluding those who have never been sexually active

**Table A1. Prior Knowledge and Screening Reported Among Populations ^A^: National Survey of Maternal and Child Health, Guatemala, 2014-2015**

|  | Guatemala (N = 15317) | | Rural (N = 8399) | | Indigenous (N = 5728) | |
| --- | --- | --- | --- | --- | --- | --- |
|  | % (SD)^B^ | N^C^ | % (SD)^B^ | N^C^ | % (SD)^B^ | N^C^ |
| **Never heard of screening** | 8.4 (0.0046) | 1266 | 12.2 (0.0072) | 970 | 17.36 (0.0104) | 972 |
| **Heard of screening and had screening** | 69.9 (0.0058) | 9660 | 66.06 (0.0084) | 4852 | 58.33 (0.0099) | 2752 |
| **Heard of screening but no screening** | 30.1 (0.0058) | 4289 | 33.94 (0.0084) | 2501 | 41.67 (0.0099) | 1924 |
| ^A^ Population has been subset to only include women aged 25 or older to match Guatemalan screening guidelines | | | | | |  |
| ^B^ Percentages and standard deviations are weighted based on DHS-provided weights | | | |  |  |  |
| ^C^ Ns are unweighted to show the relative contribution | |  |  |  |  |  |

| **Table A2. Sensitivity analysis – Prevalence ratios excluding those who have never heard of cervical cancer testing: National Survey of Maternal and Child Health, Guatemala, 2014-2015^A, B, C^** | | | | | | |
| --- | --- | --- | --- | --- | --- | --- |
|  | Guatemala | | Rural | | Indigenous | |
|  | PR | 95% CI | PR | 95% CI | PR | 95% CI |
| **Needs Permission** | 1.25 | 1.17, 1.34 | 1.27 | 1.16, 1.39 | 1.21 | 1.10, 1.33 |
| **Money** | 1.11 | 1.04, 1.18 | 1.07 | 0.99, 1.16 | 1.09 | 0.99, 1.19 |
| **Distance** | 1.08 | 1.01, 1.15 | 1.09 | 1.00, 1.18 | 1.02 | 0.93, 1.12 |
| **Doesn't want to go alone** | 1.22 | 1.15, 1.30 | 1.16 | 1.08, 1.26 | 1.10 | 1.01, 1.20 |
| **Participant language doesn't match health profession** | 1.19 | 1.1, 1.29 | 1.16 | 1.04, 1.28 | 1.21 | 1.12, 1.31 |
| ^A^ Prevalence ratios modeled using multivariate negative binomial regression with robust variance  ^B^ Models additionally adjusted for age, ethnicity, and education. | |  |  |  |  |  |
| ^C^ Population has been subset to only include women aged 25 or older to match Guatemalan screening guidelines | | | | | |  |

| **Table A3. Sensitivity analysis – Prevalence ratios excluding those who have never been sexually active: National Survey of Maternal and Child Health, Guatemala, 2014-2015^A, B, C^** | | | | | | |
| --- | --- | --- | --- | --- | --- | --- |
|  | Guatemala | | Rural | | Indigenous | |
|  | PR | 95% CI | PR | 95% CI | PR | 95% CI |
| **Needs Permission** | 1.25 | 1.17, 1.33 | 1.23 | 1.14, 1.33 | 1.20 | 1.11, 1.30 |
| **Money** | 1.09 | 1.03, 1.16 | 1.03 | 0.96, 1.11 | 1.04 | 0.97, 1.13 |
| **Distance** | 1.09 | 1.05, 1.18 | 1.09 | 1.01, 1.17 | 1.08 | 1.00, 1.17 |
| **Doesn't want to go alone** | 1.18 | 1.12, 1.25 | 1.13 | 1.06, 1.21 | 1.13 | 1.06, 1.21 |
| **Participant language doesn't match health profession** | 1.24 | 1.16, 1.33 | 1.21 | 1.11, 1.32 | 1.26 | 1.18, 1.35 |
| ^A^ Prevalence ratios modeled using multivariate negative binomial regression with robust variance  ^B^ Models additionally adjusted for age, ethnicity, and education. | |  |  |  |  |  |
| ^C^ Population has been subset to only include women aged 25 or older to match Guatemalan screening guidelines | | | | | |  |
